# The Age to Vape: Nicotine Use and Behavior Associations in Young Adults

**DOI:** 10.7759/cureus.117213

**Published:** 2026-09-30

**Authors:** Colleen Kernan, Carissa S Kwan, Elena Klyushnenkova, Niharika Khanna

**Affiliations:** 1 Family and Community Medicine, University of Maryland School of Medicine, Baltimore, USA; 2 Family Medicine, University of Maryland School of Medicine, Baltimore, USA

**Keywords:** alcohol use, cannabis use, electronic nicotine delivery systems, tobacco, young people

## Abstract

Background: Youth and young adult use of electronic nicotine delivery systems (ENDS) continues to rise. Early nicotine exposure is linked to long‑term neurobehavioral risks, and emerging evidence suggests that vaping may co‑occur with mental health and substance‑related disorders. However, limited clinical data exist on how ENDS use aligns with psychiatric and substance‑related diagnoses in young adults.

Methods: This cross‑sectional study analyzed electronic health record (EHR) data from 41,270 patients aged 18-24 years within the University of Maryland Medical System (UMMS) who completed the E‑cigarette and Vaping Assessment Tool (EVAT) between August 2020 and June 2024. ENDS use (current/former vs. never) was examined in relation to International Classification of Diseases, 10th Revision (ICD‑10) diagnoses for depressive episode (F32), anxiety disorder (F41), cannabis‑related disorder (F12), and alcohol‑related disorder (F10). Associations were assessed using chi‑square tests and multivariable ordinal logistic regression adjusting for age, sex, and race/ethnicity.

Results: Overall, 19.0% of young adults reported using ENDS. ENDS use increased with diagnostic burden, rising from 17.1% among those with no diagnoses to 58.3% among those with four diagnoses. Patients with anxiety, depression, cannabis‑related disorder, or alcohol‑related disorder consistently demonstrated higher ENDS use, with the highest prevalence (66.7%) observed among those with combined anxiety, cannabis, and alcohol‑related disorders. In adjusted models, having one or more diagnoses significantly increased the odds of ENDS use in both age groups, with the strongest effect among 18-20‑year‑olds with three diagnoses (OR ≈ 6.0).

Conclusions: ENDS use is strongly associated with psychiatric and substance‑related disorders among young adults, with risk increasing alongside the number of diagnoses. These findings underscore the need for integrated screening of vaping behaviors within mental health and substance‑use encounters, as well as concurrent assessment of behavioral health conditions among ENDS users. Embedding standardized vaping assessments into EHR workflows may support earlier identification of nicotine dependence and facilitate timely referral to cessation and addiction services.

## Introduction

Nationally, electronic nicotine delivery system (ENDS) use among youth in high school/middle school among females is 19.3%/8.2%, and any tobacco use is 23%/11.1%; in males, ENDS use is 16.6%/6.3%, and any tobacco use is 22.8%/10.9% [1-6].

According to the Monitoring the Future 2025 youth survey, vaping prevalence in 2024 was 21% among 12th graders, 15% among 10th graders, and 10% among 8th graders. In adults, the 2024 National Health Interview Survey reports that 6.9% of adults in the United States vape, while 9.9% smoke cigarettes [5,7,8].

Vaping often starts in middle and high school, driven by peer influence, curiosity, social media exposure, and coping behaviors [8]. Vaping is increasingly normalized in society, particularly among students experiencing stress, anxiety, or depression, and may co-occur with the use of cannabis [7]. Early nicotine exposure poses significant risks to adolescent brain development, with effects that persist into adulthood [1,3,4,9,10]. A 2020 study of Leslie [4] notes that the behavioral changes triggered by nicotine use in adolescents carry over into adulthood, including mood changes, decreased attention span, and an increased reward effect from other abused drugs. These findings raise concerns that vaping may co-occur with nicotine, cannabis, alcohol, and other substance use [11,12].

Concerning trends, little is known about how vaping co-occurs with mental health and substance-related disorders across young adults. Adverse childhood experiences, including abuse, caregiver incarceration, and household substance misuse, further elevate the risk of tobacco and nicotine use, underscoring the complex interplay between behavioral health and nicotine exposure [7].

To address this gap, we analyzed electronic health record (EHR) data from the University of Maryland Medical System (UMMS) using a standardized vaping assessment tool. Our study evaluates whether the likelihood of ENDS use increases with the number of diagnosed behavioral health and substance use disorders among young adults.

## Materials and methods

Study population

The UMMS is a university-based regional healthcare system that provides primary, specialty ambulatory, and inpatient care at 11 hospitals and 150+ ambulatory locations, forming one of the largest healthcare systems in the state of Maryland.

Our team facilitated the implementation of a comprehensive, EHR-based E-cigarette and Vaping Assessment Tool (EVAT) within the UMMS EHR in August 2020, providing discrete choices for documenting vaping status. Data for this study were retrieved from the UMMS Epic EHR for 41,270 patients. To be included in the analysis, a patient must (a) have been evaluated using EVAT between August 2020 and June 2024 and (b) be between the ages of 18 and 24 years old. For patients evaluated several times during the study period, the most recent status was used. Patients were excluded if demographic data (age, sex, race/ethnicity) in their EHR history were missing. These data were collected across the UMMS and represent the catchment area across 14 counties in Maryland. Further, we used the International Classification of Diseases, 10th Revision (ICD-10) codes to ensure accuracy of diagnosis and to decrease biases introduced by self-report.

Variables

EHR data on ENDS use, demographics (age, sex, race/ethnicity), and ICD-10 codes for medical diagnoses were extracted. ENDS use was determined from EVAT and divided into two mutually exclusive categories: ever used ENDS and never used ENDS. Any instance of ENDS use was considered a positive response. Diagnoses for depressive episode (code F32), anxiety disorder (code F41), cannabis-related disorder (code F12), and alcohol-related disorder (code F10) were based on ICD-10 codes for medical problems at the time of EVAT assessment. Any instance of the ICD-10 code of interest (current or past) that was present in the patient's EHR at the time of EVAT assessment was considered a positive response. Patients were categorized into 16 different possible combinations of diagnoses, as well as by the number of diagnoses they had. Age, sex, and race/ethnicity were included as covariates. Age was categorized as 18-20 and 21-24 years old. Race/ethnicity was categorized as White non-Hispanic or unknown ethnicity, Black or African American non-Hispanic or unknown ethnicity, and Other or unknown race and ethnicity.

Statistical analysis

Descriptive statistics for the study sample were calculated, with frequency and percent reported. A breakdown of patients who have depressive episode, anxiety disorder, cannabis-related disorder, and/or alcohol-related disorder was included. Chi-square tests for significance of association between ENDS use and the number of diagnoses were analyzed for the full sample and by age strata. The association between ENDS use and the number of diagnoses was further analyzed using a multivariable nominal logistic regression model that included adjustment for covariates and interactive effects of age and number of diagnoses. Statistical significance for all tests was set at α = 0.05 (two-tailed test), and all analyses were computed using SAS version 9.4 (SAS Institute Inc., Cary, NC).

Ethics statement

This study was designated as exempt by the University of Maryland Baltimore Institutional Review Board (reference number: HP-00093456).

## Results

The UMMS is a Maryland-based health system affiliated with a public university serving a high proportion of underserved patients. Results presented here are from 41,270 patients aged 18-24 years seen at the UMMS between August 2020 and June 2024. All data were extracted from the Epic EHR and utilized an e-cigarette/vaping assessment tool that had been developed by this team within the EHR.

Population characteristics

Frequencies and proportions for demographic characteristics, ENDS use, and the four targeted ICD-10 code medical diagnoses are shown in Table 1. Among 41,270 patients in the study sample, 7,851 (19.0%) ever used (current and former users) ENDS, 4,179 (10.1%) had been diagnosed with depressive episode, 5,812 (14.1%) had been diagnosed with anxiety disorder, 1,039 (2.5%) had been diagnosed with cannabis-related disorder, and 420 (1.0%) had been diagnosed with alcohol-related disorder. A total of 14,993 (36.3%) patients were aged 18-20, and 26,277 (63.7%) were aged 21-24. Additionally, 63.3% of the patients were female, 46.4% were non-Hispanic White, and 32.6% were non-Hispanic Black.

**Table 1:** Demographic and clinical characteristics for 41,270 young adult UMMS patients evaluated for ENDS use through EVAT between August 2020 and June 2024. ENDS: electronic nicotine delivery systems; EVAT: E‑cigarette and Vaping Assessment Tool; UMMS: University of Maryland Medical System

Characteristic		n (%)
Depressive episode	Yes	4,179 (10.1)
Anxiety disorder	Yes	5,812 (14.1)
Cannabis-related disorder	Yes	1,039 (2.5)
Alcohol-related disorder	Yes	420 (1.0)
ENDS use	Yes	7,851 (19.0)
Age, years	18-20	14,993 (36.3)
	21-24	26,277 (63.7)
Sex	Female	26,110 (63.3)
	Male	15,160 (36.7)
Race/Ethnicity	Non-Hispanic White	19,139 (46.4)
	Non-Hispanic Black	13,459 (32.6)
	Other	8,672 (21.0)

Sixteen possible different combinations resulting from the four diagnoses of interest (depressive episode, anxiety disorder, cannabis-related disorder, and alcohol-related disorder) are shown in Table 2, overall and by age strata. A total of 32,724 (79.3%) patients did not have any of the four diagnoses, with 12,268 of them 18-20 years old and 20,456 of them 21-24 years old. The highest diagnosis combination is anxiety disorder, with 6.8% (N = 1,025) of 18- 20-year-olds diagnosed and (N = 2,330) 8.9% of 21- 24-year-olds diagnosed. The lowest diagnosis combination is anxiety disorder, cannabis-related disorder, and alcohol-related disorder, with <0.1% of patients (ages 18-20: 2; ages 21-24: 4) diagnosed.

**Table 2:** Prevalence of diagnoses by age group for 41,270 young adult UMMS patients evaluated for ENDS use through EVAT between August 2020 and June 2024. ENDS: electronic nicotine delivery systems; EVAT: E‑cigarette and Vaping Assessment Tool; UMMS: University of Maryland Medical System

Number of Diagnoses	Combination of Diagnoses	All (N=41,270)	Age, Years	
			18-20 (n=14,993)	21-24 (n=26,277)
		n (column %)	n (column %)	n (column %)
No Diagnoses	None	32,724 (79.3)	12,268 (81.8)	20,456 (77.9)
1 Diagnosis	Depression	1,686 (4.1)	630 (4.2)	1,056 (4.0)
	Anxiety	3,355 (8.1)	1,025 (6.8)	2,330 (8.9)
	Cannabis	558 (1.4)	169 (1.1)	389 (1.5)
	Alcohol	248 (0.6)	67 (0.5)	181 (0.7)
2 Diagnoses	Depression, Anxiety	2,120 (5.1)	665 (4.4)	1,455 (5.5)
	Depression, Cannabis	144 (0.4)	49 (0.3)	95 (0.4)
	Depression, Alcohol	42 (0.1)	10 (0.1)	32 (0.1)
	Anxiety, Cannabis	142 (0.3)	50 (0.3)	92 (0.4)
	Anxiety, Alcohol	26 (0.1)	6 (<0.1)	20 (0.1)
	Cannabis, Alcohol	32 (0.1)	7 (0.1)	25 (0.1)
3 Diagnoses	Depression, Anxiety, Cannabis	121 (0.3)	29 (0.2)	92 (0.4)
	Depression, Anxiety, Alcohol	30 (0.1)	3 (<0.1)	27 (0.1)
	Depression, Cannabis, Alcohol	24 (0.1)	10 (0.1)	14 (0.1)
	Anxiety, Cannabis, Alcohol	6 (<0.1)	2 (<0.1)	4 (<0.1)
4 Diagnoses	Depression, Anxiety, Cannabis, Alcohol	12 (<0.1)	3 (<0.1)	9 (<0.1)

The prevalence of ENDS use within each of the 16 diagnosis combinations is shown in Table 3. Patients who do not have any diagnoses have the lowest percentage of ENDS use (17.1%), while those who have a combination of anxiety disorder, cannabis-related disorder, and alcohol-related disorder have the highest percentage of ENDS use (66.7%).

**Table 3:** Prevalence of ENDS use by diagnoses for 41,270 young adult UMMS patients evaluated for ENDS use through EVAT between August 2020 and June 2024. ENDS: electronic nicotine delivery systems; EVAT: E‑cigarette and Vaping Assessment Tool; UMMS: University of Maryland Medical System

Number of Diagnoses	Combination of Diagnoses	ENDS Use	
		Yes	No
		n (row %)	n (row %)
No Diagnoses	None	5,601 (17.1)	27,123 (82.9)
1 Diagnosis	Depression	416 (24.7)	1,270 (75.3)
	Anxiety	752 (22.4)	2,603 (77.6)
	Cannabis	191 (34.2)	367 (65.8)
	Alcohol	110 (44.4)	138 (55.7)
2 Diagnoses	Depression, Anxiety	505 (23.8)	1,615 (76.2)
	Depression, Cannabis	50 (34.7)	94 (65.3)
	Depression, Alcohol	19 (45.2)	23 (54.8)
	Anxiety, Cannabis	69 (48.6)	73 (51.4)
	Anxiety, Alcohol	14 (53.9)	12 (46.2)
	Cannabis, Alcohol	19 (59.4)	13 (40.6)
3 Diagnoses	Depression, Anxiety, Cannabis	64 (52.9)	57 (47.1)
	Depression, Anxiety, Alcohol	17 (56.7)	13 (43.3)
	Depression, Cannabis, Alcohol	13 (54.2)	11 (45.8)
	Anxiety, Cannabis, Alcohol	4 (66.7)	2 (33.3)
4 Diagnoses	Depression, Anxiety, Cannabis, Alcohol	7 (58.3)	5 (41.7)

Associations between ENDS use and number of diagnoses

In bivariate analyses, the number of diagnoses, age, sex, and race/ethnicity were all significant with ENDS use (Table 4). In addition, ENDS use was noted in a higher proportion of patients who are aged 21-24 (N = 5,248; 20.0%), are male (N = 3,461; 22.8%), or are non-Hispanic White (N = 5,081; 26.6%), compared to other groups within the same demographic categories. The percentage of patients using ENDS increases as the number of diagnoses increases, with only 17.1% (N = 5,601) of patients with zero diagnoses using ENDS compared to 58.3% (7) of patients with four diagnoses using ENDS (Table 4). The effect of the number of diagnoses on ENDS use was further assessed in a multivariable nominal logistic regression model, accounting for age as an effect modifier between the main predictor and outcome. Adjusted odds ratios (OR) comparing ENDS users to those who never used ENDS in age strata, and controlling for sex and race/ethnicity, are shown in Table 5. ORs for association between ENDS use and other demographic characteristics derived from the same multivariable regression model are shown in Table 6. Due to paucity of data regarding patients with all four diagnoses, these results may not be accurately reflective of the population and have been suppressed in Tables 5-6.

**Table 4:** Association between ENDS use and demographic and clinical characteristics for 41,270 young adult UMMS patients evaluated for ENDS use through EVAT between August 20 and June 2024. ENDS: electronic nicotine delivery systems; EVAT: E‑cigarette and Vaping Assessment Tool; UMMS: University of Maryland Medical System

Characteristic		ENDS Use		χ2 (p)
		Yes	No	
		n (row %)	n (row %)	
Number of diagnoses	0	5,601 (17.1)	27,123 (82.9)	478.3868 (<0.0001)
	1	1,469 (25.1)	4,378 (74.9)	
	2	676 (27.0)	1,830 (73.0)	
	3	98 (54.1)	83 (45.9)	
	4	7 (58.3)	5 (41.7)	
Age, years	18-20	2,603 (17.4)	12,390 (82.6)	42.2275 (<0.0001)
	21-24	5,248 (20.0)	21,029 (80.0)	
Sex	Female	4,390 (16.8)	21,720 (83.2)	225.3647 (<0.0001)
	Male	3,461 (22.8)	11,699 (77.2)	
Race/Ethnicity	Non-Hispanic White	5,081 (26.6)	14,058 (73.5)	1312.0302 (<0.0001)
	Non-HispanicBlack	1,669 (12.4)	11,790 (87.6)	
	Other	1,101 (12.7)	7,571 (87.3)	

**Table 5:** Association between ENDS use and the number of diagnoses by age groups for 41,270 young adult UMMS patients evaluated for ENDS use through EVAT between August 2020 and June 2024. These are the results of the multivariable nominal logistic regression model. ENDS: electronic nicotine delivery systems; EVAT: E‑cigarette and Vaping Assessment Tool; UMMS: University of Maryland Medical System

Number of Diagnoses (ref = 0)	ENDS Use (ref = Never Used)	
	aOR (95% CI)	Pr > |z|
Ages 18-20		
1 Diagnosis	1.709 (1.519, 1.923)	<0.0001
2 Diagnoses	1.849 (1.563, 2.187)	<0.0001
3 Diagnoses	6.049 (3.284, 11.142)	<0.0001
Ages 21-24		
1 Diagnosis	1.422 (1.310, 1.543)	<0.0001
2 Diagnoses	1.515 (1.350, 1.700)	<0.0001
3 Diagnoses	4.882 (3.447, 6.914)	<0.0001

**Table 6:** Association between ENDS use and number of diagnoses by age groups for 41,270 young adult UMMS patients evaluated for ENDS use through EVAT between August 2020 and June 2024. These are the full results of the multivariable nominal logistic regression model. --: It is an exact value, and no confidence interval is needed, as 0 Diagnoses is used as the reference point. ENDS: electronic nicotine delivery systems; EVAT: E‑cigarette and Vaping Assessment Tool; UMMS: University of Maryland Medical System

Characteristics		ENDS Use (ref = Never Used)	
		aOR (95% CI)	aOR (95% CI)
Number of diagnoses by age (years)			
18-20	0 Diagnoses	1.000 (--, --)	--
	1 Diagnosis	1.709 (1.519, 1.923)	<0.0001
	2 Diagnoses	1.849 (1.563, 2.187)	<0.0001
	3 Diagnoses	6.049 (3.284, 11.142)	<0.0001
21-24	0 Diagnoses	1.000 (--, --)	--
	1 Diagnosis	1.422 (1.310, 1.543)	<0.0001
	2 Diagnoses	1.515 (1.350, 1.700)	<0.0001
	3 Diagnoses	4.882 (3.447, 6.914)	<0.0001
Other demographic characteristics			
Sex	Female	1.000 (--, --)	--
	Male	1.491 (1.416, 1.569)	<0.0001
Race/ethnicity	NH White	1.000 (--, --)	--
	NH Black	0.410 (0.386, 0.436)	<0.0001
	Other	0.430 (0.400. 0.462)	<0.0001

Among the 18-20-year-olds, patients with one, two, or three diagnoses had significantly higher odds of ENDS use compared to those who had no diagnoses. Among 21-24-year-olds, patients who had any number of diagnoses had significantly higher odds of ENDS use compared to those in the same age strata with no diagnoses. For both age groups, the odds of ENDS use were not significantly different for patients with only one diagnosis compared to patients with two diagnoses. Patients with three diagnoses had significantly higher odds of ENDS use compared to those with one or two diagnoses, indicated by the higher, non-overlapping 95% CIs (ages 18-20: (3.284, 11.142); ages 21-24: (3.447, 6.914), Table 6)).

## Discussion

National data suggest that use of all substances is high in individuals with behavioral health diagnoses. Our data suggest that vaping rates are high in young adults, and the use of nicotine may co-occur with cannabis and alcohol use in the presence of anxiety and depression. Our analysis reveals a clear and consistent association between ENDS use and psychiatric and substance-related disorders among youth and young adults. A significantly higher prevalence of ENDS use is noted across all four categories, namely, depressive episode, anxiety disorder, cannabis-related disorder, and alcohol-related disorder, compared to those without such diagnoses. Substance-related conditions, particularly alcohol and cannabis disorders, showed the strongest associations with ENDS use, highlighting the potential for multi-substance use behaviors. Nearly half of patients with alcohol-related disorder were ENDS users, while more than 40% with cannabis-related disorder were ENDS users. These findings are consistent with the observations of others in the published literature; that is, anxiety and depression may co-exist with multi-substance disorders [13-15].

These patterns of vaping may raise concerns that vaping may precede or be concurrent with other substance use, but this study did not directly assess causality. The observed high rates of vaping in young adults may suggest renormalization of nicotine use with negative consequences on health [13-16]. Early initiation of nicotine exposure has been linked to increased dependence severity, increased likelihood of future cigarettes and other tobacco use, and greater likelihood of sustained substance use disorders later in adulthood, underscoring the long-term public health relevance of these findings [16,17].

Our findings agree with the literature by demonstrating higher ENDS use among non-Hispanic White patients (26.6%) and males (22.8%), while rates among non-Hispanic Black and Other racial/ethnic groups were substantially lower. These patterns underscore the importance of tailoring prevention strategies to each demographic group [6,16]. The likelihood of ENDS use increased with the number of diagnoses in both age groups, with the strongest effect among younger patients aged 18-20 with three diagnoses (OR ≈ 6.0) compared to older patients aged 21-24 years (OR ≈ 4.9). The strong association between ENDS use and mood or anxiety disorders may suggest that vaping may serve as a coping mechanism, reinforcing the need for integrated mental health screening [18-20].

Routine screening for vaping behaviors during psychiatric and substance-related encounters could enable early identification and intervention for nicotine dependence. Given the strong co-occurrence of ENDS use with mental health and substance-related diagnoses, routine assessment of vaping behaviors during psychiatric and substance-related encounters and during annual checkups may facilitate early identification of nicotine dependence and related risk. Similarly, youth reporting ENDS use should concurrently be screened for depression, anxiety, and substance-related disorders. Integrating such screening protocols within EHR systems can aid in streamlining identification of such disorders and promote timely referrals to tobacco cessation services, such as state quit lines, and to multi-substance addictions management.

This study is not without limitations. First, the cross-sectional nature of the available EHR data prevents determination of causal relationships. Second, ENDS use was documented via clinical assessment and patient self-report rather than objective verification, which may underestimate true prevalence due to underreporting or incomplete documentation. Third, ICD-10 diagnoses capture only clinically recognized conditions, potentially missing subthreshold or untreated mental health and substance use symptoms. Fourth, some stratified subgroup analyses were limited by small cell sizes, yielding unstable prevalence estimates in certain categories, including several values approaching 100%. Lastly, this study did not have longitudinal assessments in the study design to assess causality.

Despite these limitations, the strengths of this study include its large, diverse clinical sample; use of a standardized EHR screening tool embedded in routine care; detailed stratification by age, sex, and race/ethnicity; and the use of ICD-10 diagnosis [21]. Collectively, the results demonstrate clear and consistent associations between ENDS use and psychiatric and substance-related conditions among young adults. Future longitudinal studies could examine causality and trajectories of co-use over time, evaluate whether early mental health interventions reduce vaping initiation, and assess the effectiveness of integrated cessation strategies within mental health and substance use treatment settings. Given the growing burden of both youth vaping and mental health disorders, coordinated clinical and public health responses across primary and specialty care are urgently needed to mitigate long-term harms.

## Conclusions

In this large, diverse cohort of young adults within a major Maryland health system, ENDS use was consistently and strongly associated with depressive episodes, anxiety disorders, and cannabis‑ and alcohol‑related disorders. Vaping prevalence rose sharply with increasing diagnostic burden, with patients who had multiple co‑occurring conditions showing the highest likelihood of ENDS use.

These findings highlight the importance of integrating routine vaping assessments into mental health and substance‑use encounters, as well as screening ENDS users for underlying psychiatric or substance‑related conditions. Embedding standardized tools such as EVAT into clinical workflows can support earlier identification of nicotine dependence and facilitate timely referral to cessation and addiction services. Although the cross‑sectional design limits causal inference, the strong associations observed emphasize the need for longitudinal research to clarify temporal patterns of co‑use and to evaluate whether early behavioral health interventions can reduce vaping initiation or progression. Coordinated clinical and public health strategies are urgently needed to address the intertwined burdens of youth vaping, mental health disorders, and substance use.
